# Biofeedback Therapy for Stress Urinary Incontinence: Is Urodynamic Assessment Necessary?

**DOI:** 10.5812/numonthly.2835

**Published:** 2012-06-20

**Authors:** Narihito Seki, Nouval Shahab

**Affiliations:** 1 Department of Urology, Graduate School of Medical Sciences, Kyushu University, Fukuoka, Japan

**Keywords:** Urinary Incontinence, Biofeedback, Psychology

Dear Editor

In the recent issue of Nephro-Urology Monthly Journal, Bayrak et al. (1) presented a prospective, single institutional study to evaluate the effects of biofeedback therapy (BFT) on stress urinary incontinence (SUI) in women. The International Consultation on Incontinence Questionnaire – Short Form (ICIQ-SF) was utilized and ICIQ-SF scores were measured to evaluate the severity of their urinary incontinence symptoms. In addition, urodynamic investigation was performed and its parameters were examined. The results of this study demonstrated the remarkable improvement of ICIQ-SF scores following BFT while urodynamic parameters were not affected. The authors concluded that BFT is a good alternative to surgical treatment of SUI and urodynamic assessment is not recommended prior to BFT, as the values of urodynamic parameters did not change following BFT. The subject matter of this study is important and highly relevant as BFT has been intensively studied in women with urinary incontinence. Its potential efficacy has also been suggested in men, (2) and even in children (3) with urinary incontinence.

A previous Cochrane systematic review and meta-analysis study (4) that critically evaluated 12 randomized control trials in the management of female urinary (stress, urge, and mixed) incontinence, demonstrated that pelvic floor muscle training (PFMT) alone gave better improvement of symptoms and continence specific quality of life, when compared to other treatments (no treatment, placebo, sham treatment, or other inactive control treatments), particularly in women with SUI alone, who seemed to have a greater treatment effect. In the management of SUI, BFT is a common adjunct used along with pelvic floor muscle training (PFMT) to improve training performance. Previous evidence also supported the possibility that BFT may provide benefits in addition to PFMT in women with urinary incontinence, the question remains as to what will be the impact of this study in supporting the benefits of using BFT for SUI. With respect to the important messages delivered, the methodology utilized requires critical appraisal in the absence of treatment control. Therefore, the improvement of ICIQ-SF may not only be affected by BFT, but may also be influenced by other behavioral treatments.

Regardless of its limitations, the results support what has been demonstrated in other previous studies, as summarized by Herderschee et al. (5) in a more recent Cochrane systematic review and meta-analysis study. Among the 17 control trials and contributing data that was evaluated, they concluded that BFT in addition to PMFT may provide benefits in women with urinary incontinence. Nevertheless, further research is required as suggested by the authors, to differentiate whether the BFT, or some other differences between the trials, such as more contact with health professionals caused the beneficial effects.

Unnecessary urodynamic evaluation prior to BFT as suggested in this study, is also questionable, if only based on this trial. As a matter of fact, urodynamic study is not routinely used prior to conservative or non-surgical treatment for SUI. Recently, urodynamic investigation has become controversial even for use prior to the surgical treatment of SUI. The inconclusive results regarding its ability to predict the outcome following surgery, due to the limited number of good clinical evidence, was considered to be one of the reasons.

In contrast to the unchanged urodynamic parameters (bladder capacity, detrusor instability, sensation of the bladder, and residual urine volume) following BFT as reported in this trial, Capelini et al. (6) observed a significant increase in Valsalva leak point pressure, cystometric capacity and bladder volume at first desire to void following BFT. However, only a small number of patients (14) with SUI were included and accordingly, that may decrease the study’s reliability. Although both studies applied different types of validated questionnaire (ICIQ-SF versus King’s Health Questionnaire), the improvement in symptoms was consistent in both studies. As only a few studies have reported the effect of BFT on voiding performance, which focused on urodynamic assessment, this study represents a valuable and noteworthy addition to the field. Moreover, this trial also highlights an important point regarding the efficacy of BFT in patients who bordered on obese. The present trial and previous series provide us with valuable evidence that has rarely been found in other series.

## References

[1] Bayrak O, Seckiner I, Erturhan MS, Erbagci A, Yagci F (2011). The effect of biofeedback therapy on ICIQ-SF scores and urodynamic parameters in patients with stress urinary incontinence. Nephro-Urol Mon.

[2] Goode PS, Burgio KL, Johnson TM, 2nd, Clay OJ, Roth DL, Markland AD (2011). Behavioral therapy with or without biofeedback and pelvic floor electrical stimulation for persistent postprostatectomy incontinence: a randomized controlled trial. JAMA.

[3] Yagci S, Kibar Y, Akay O, Kilic S, Erdemir F, Gok F (2005). The effect of biofeedback treatment on voiding and urodynamic parameters in children with voiding dysfunction. J Urol.

[4] Dumoulin C, Hay-Smith J (2010). Pelvic floor muscle training versus no treatment, or inactive control treatments, for urinary incontinence in women. Cochrane Database Syst Rev.

[5] Herderschee R, Hay-Smith EJ, Herbison GP, Roovers JP, Heineman MJ (2011). Feedback or biofeedback to augment pelvic floor muscle training for urinary incontinence in women. Cochrane Database Syst Rev.

[6] Capelini MV, Riccetto CL, Dambros M, Tamanini JT, Herrmann V, Muller V (2006). Pelvic floor exercises with biofeedback for stress urinary incontinence. Int Braz J Urol..

